# Paresis, or General Paralysis of the Insane

**Published:** 1883-07

**Authors:** Wm. D. Granger

**Affiliations:** First Assistant Physician, State Asylum for the Insane, Buffalo, N. Y.


### Paresis, or General Paralysis of the Insane.*

By Wm. D. Granger, M. D.,

First Assistant Physician, State Asylum for the Insane, Buffalo, N. Y.

We shall, in this paper, dwell mostly upon the practical aspects of paresis, and consider the natural and clinical history of the disease, and the diagnostic symptoms, especially the earlier stages, when the patient is most apt to come under the observation of the general practitioner.

The disease has been recognized and described within the limits of this century. The first cases in this country were reported in 1849. Is paresis, therefore, to be considered one of the "new and rare" diseases that would come within the limits of Sir James Paget's definition, as made in his recent Bradshawe lecture, namely, that there are facts enough "not, indeed, to prove, but to justify the belief that we have here an example of disease which has appeared in this country for the first time within the limits of the last century, and which has since become sufficiently frequent and acquired sufficient and distinctive characters to be described by a general term and be called by a new name."

This is an interesting question, but cannot be fully discussed to-night. The disease is, however, one of localities, and is almost wanting in some countrys, or even parts of the same country. In America it occurs more frequently in the Northern and Eastern States. At the Utica asylum, up to 1881 there had been 439 cases of paresis admitted. In that year one in nine of the men admitted was a paretic. At the Buffalo asylum, of 492 admissions, thirty had paresis, while last year one in eight of the men had that form of disease. In the Louisiana State asylum there was no case of paresis among the 127 patients in their last report; while at the Wisconsin State asylum, out of 683 patients, but four had the disease. Why this difference it is difficult, nay impossible, with our pres-

* Read before Buffalo Medical and Surgical Association, Feb. 6th, 1883.

ent knowledge, to tell. There is much to cause us to believe that it is a new disease, and that it appears in different localities, where its progress may be formulated by the following law: The appearance of the disease among males, its increase among males, its appearance among females, and its increase slowly among them, then a stationary condition, when the disease seems to bear a constant proportion to the total cases admitted to an asylum.

There is much to interest and instruct in studying the etiology of paresis. The disease is found largely, but not exclusively, among men of middle life, strong, active and robust, among those of good circumstances and surroundings, rather than the ignorant, the poor or the laborer. But if the disease exists largely among this class, it is also true that those who have the disease are very frequently given to excess in the use of liquor, or they have suffered from syphilis, or they are known to have indulged in venereal excess. Often all these conditions have existed together and formed a part of the life of the paretic. For a fast young man to have paresis is a common matter, but for a virtuous young woman to have it is extremely rare, if it ever occurs. Of six women admitted to the Buffalo asylum, with paresis, one was married, two had been married twice, one being an actress and had led a wandering life, and three were prostitutes. I would express no stronger opinion than this, that paretics are largely found among the class described, and that syphilis and the other conditions mentioned are very frequently a part of the history of those having the disease. Cases not infrequently occur where none of these conditions are present.

In approaching the study of the clinical history of paresis, it is to be observed that in every case certain conditions are present, namely, there must be paralysis, there must be insanity, and the final result must be death. But remember the condition is not simply insanity plus paralysis, but that the form of both the insanity and paralysis is peculiar to this disease, and that they are associated together in a manner unlike any other manifestations of these conditions.

An effort to define paresis is rather an unsatisfactory task, but it will aid in further discussing the subject to attempt it at this place. It is a disease characterized by an association of mental and physical symptoms, of which progression is the prominent quality. Physical symptoms are marked by progressive, incomplete paralysis of the muscles, especially those of speech and locomotion. The mental symptoms show a tendency towards dementia, and generally there are extravagant delusions, progressive, also, in grandeur and variety. This definition is a combination of several of the best that have been given. I would add, that in my opinion no definition is complete without saying that it is further characterized by so-called "paretic seizures," progressive, also, in their severity, and which partake of the nature of apoplectic, paralytic or epileptic attacks.

Paresis is divided into stages, but as it is a progressive disease, these are not always well marked. For our purpose it is best to consider three stages, which will cover the history of the full development of the disease, and make the so-called prodromic period a part of the first stage. The prodromic symptoms often continue a somewhat prolonged time before the full onset of the disease. They are not, as a rule, recognized by the friends, and if recalled at all, it is after memory is quickened by the questions of the physician. Among the earlier symptoms is perversion of the moral sense. If the patient is a moral man, violations of decency, debauchery, or even open immorality may occur, or if he is a man of loose habits, he becomes even more sensual. Petty thefts occur, not infrequently, early in the disease. A case came under the observation of the writer, of a clerk who stole the dinner pails of working men connected with the establishment. Great irritability, sudden passion, violent, threatening and even profane language in those who never gave way to anger, or increased want of self-control in those naturally lacking in those qualities characterize this stage.

There is early noticed, or rather, remembered, increased activity but want of steadiness of purpose; expansion of ideas and a tendency to speculation, change of manner and perhaps loudness of dress, boastfulness and failure of judgment. All of this sums of briefly a change of character, which gradually gives anxiety to friends, but does not make them consider disease of the brain as a cause for this change, and therefore they do not consult a physician. Sometimes there is depression, gloomy fears, failure of memory, and perhaps seclusive habits, and sometimes the patient early consults a physician. Physical symptoms frequently appear at this time. There is sleeplessness and severe headache, perhaps some neuralgic pains. There may be flushing of the face, a sense of fullness of the head, or severe attacks of cerebral congestion, with periods of confusion of ideas, or a dazed or stunned condition, when the patient may need, for a little while, the assistance of bystanders, and followed by a feeling of exhaustion, and perhaps tingling in the fingers, or numbness in them. Possibly there may be a slight defect in speech for a day or two. These attacks are paretic seizures in their simplest form. These early symptoms are more or less pronounced, and gradually lapse into the first stage of the confirmed appearance of the disease.

If at this time one's attention is called to a paretic, it would be a bad error to fail to appreciate the gravity of the symptoms and the probability of the presence of the disease, with warning words to the friends of the patient, and close watchfulness on our part. It is impossible to make the patient appreciate his condition.

As the disease advances there is an increase of the symptoms already spoken of. There is greater expansion of ideas, greater willfulness and self-contentment, a stronger determination of purpose with an ever-changing end in view, and an unwillingness to be guided or controlled by others.

In studying the symptoms of this stage, it must be remembered that they are both physical and mental, and that they are also progressive. It must also be remembered that they do not always go hand in hand; nor is the progression continuous and constant; and that remissions may occur, so marked in some cases as to make those unacquainted with the disease suppose it is a recovery. It is an interesting question, and by no means a settled one, whether the physical symptoms first occur, or the mental. The mental changes, most frequently, are first noticed, probably because the attention is more easily attracted by them than by the minute symptoms first marking the physical changes; sometimes the physical symptoms are advanced while the mental symptoms are slow in appearing, and only a general sense of well-being, a want of appreciation and care for one's condition, and other slight changes of character, attract attention. On the other hand, the mental symptoms may be well developed while the physical are to be discovered by careful examination only.

In the earlier period of the first stage, the physical or paretic symptom, most constant and always to be looked for, is a slight change of speech. It may be so slight as to be noticed only by the accustomed ear; in its slightest form, it is simply the slurring of a word. As the difficulty advances there is a thickness of speech, a hesitating at certain words, and then their explosion. Frequently a word or syllable is left out or repeated. When plainly to be noticed, the speech is much like that of a drunken man. In cases of marked remission, a slight remnant of this defect of speech is generally to be noticed. If while the patient is speaking, or if he protrudes his tongue, you will frequently notice a peculiar twitching of skin and soft parts about the mouth, particularly at the corners and alongside the nose, and also a tremulous vernacular-like movement to the tongue. It may with difficulty be protruded and quickly drawn back. This ataxic condition may be very slight, and a good light and prolonged and careful examination, with a knowledge of just what to look for, is often necessary in order to discover slight changes. Sometimes these symptoms closely resemble the appearance of the mouth of a person when about to weep. In more advanced cases, this tremulousness and twitching may extend to the whole face, and be plainly marked when it is in repose.

Inequality of the pupils is often noticed, one pupil being largely dilated.

The gait frequently shows change, there being the same want of co-ordination of the muscles of the extremities as are found in the face. If asked to walk, the patient will make an effort to do his best, but he soon shows peculiarities; the feet are somewhat separated and the gait may be called "wide;" he handles them carefully and places them on the floor with an effort to steady himself. As the disease progresses, he takes wide turns in going around corners, or if he stops suddenly, has to put out his hands to steady himself. The difficulty of walking gradually increases and the patient walks with outstretched hands, stumbling along and shuffling his feet. In the hands the fine movements of the fingers requiring co-ordination are lost. If a musician, the niceness to touch; if a mechanic, the ability to handle tools. The handwriting becomes altered; it is irregular, scratchy and angular; as in speech, words or syllables are left out or repeated.

If the mental symptoms keep pace with the physical, there is noticed greater activity; the patient is up early and goes to bed late; he walks the streets all day, intent upon business, he tells you, though really he does nothing. He becomes more boastful, perhaps speaks of increased wealth or that his next ventures will bring him much money. He is unwilling to be controlled, and perhaps becomes very violent if any attempt is made. He is contented and happy, and will tell you he is "first-rate," that he was never better in his life. Ideas of personal grandeur fill the paretic's brain. He is the strongest or most learned of men. His delusions are mostly self-centered, and if any one partakes of his favor they are bestowed by the paretic and become a part of his glory.

If, as the disease progresses, he is God, he makes his attendants angels, or if a king, nobles.

Sometimes, as has been said, marked self-contentment and feeling of well-being alone characterize the disease, with ever increasing dementia; and extravagant delusions, if they exist, have to be found by questioning the patient. Sometimes the history of paresis is that of a mental condition resembling melancholia, and they may even commit suicide. There are still present, the exaggerated ideas of the paretic, and relate wholly to himself. A melancholic paretic, now at the Buffalo asylum, thinks he is the devil, and has become so from his great wickedness. Sometimes they are cross, irritable and sullen, and treat all who approach them with haughty disdain.

The second stage is an exaggeration of the first. It may be passed quickly over and the patient sink into the dementia of the third stage, or it may be much prolonged, with remissions or stationary periods where there is little advance of the disease. Often great maniacal violence exists in this stage, but he is generally happy and contented. He shares his wealth and good fortune with his fellow-patients, and, if he feels the restraint of the asylum, is good natured, for he will say, "I am going home to-morrow." Often there is voracious appetite; and the food is stuffed into the mouth so that deaths occur from strangulation.

As the disease progresses and passes into the third stage,that of extreme paretic symptoms and advanced dementia, the delusions grow in magnitude, especially of his personal powers and greatness, while the increasing paralysis makes the boast and the reality only the more marked. If the patient lives long enough, the dementia approaches, as near as possible, to a condition of amentia: unable to stand or walk, without any control over the sphincters, his life is wretched to all but himself: if he can give any evidence, it is that he is happy. Bed sores and extensive sloughing often take place; death may occur from gradual failure of vital powers or from inter-current disease, but most often during a paretic seizure. These seizures now demand attention and deserve careful consideration. They occur in almost every case; It has never come within my observation to see a case where in some form it did not occur. They are way-marks in the history of a patient. Sometimes they are the first symptoms that really excite apprehension in the minds of friends. Their occurrence generally terminates a remission and ushers in the final end, and death frequently takes place during one of them. They are, as has before been said, either apoplectic, paralytic or epileptiform in character. In their simplest forms they often occur so early in the disease as to escape observation. If questioned, the friends will remember a day when the patient complained, in the morning, of weakness in the legs, and felt tired and remained quiet in the house for a day or two, or his speech was a little changed. If called at this time, examine for the ataxic symptoms of the face, for they are apt to be more pronounced. There may be a history of attacks resembling *petit mal*, and the patient complaining of being dizzy, will find locomotion impeded for several hours. If, with such attacks, there appear mental and physical symptoms as have been described, never mind how slight, one can hardly do wrong to diagnosticate paresis. As the disease advances, the severity of these attacks generally increases, and their appearance coincides with increase all the symptoms, sometimes life is fortunately ended in the second stage, by a severe apoplectic-like seizure. As the end approaches the attacks become more severe. When epiliptiform convulsions occur they are sometimes most violent, and as many as a hundred may rapidly follow one another, before death takes place. The average length of the disease is about three years; sometimes it lasts two or three times as long, and in some cases but a few months after the full appearance of the symptoms.

In making a diagnosis, always bear in mind epilepsy with mania or dementia; also, ordinary paralysis with the different forms of insanity, especially paralysis and dementia.

Ordinary acute mania with extravagant delusions must be remembered, and sometimes a diagnosis for a time withheld. If, however, one always keeps in mind the peculiar association of ataxic and mental symptoms, carefully examines his patient and gets a good history from the friends, there is little danger of drawing wrong conclusions, though for a time it may be impossible to pronounce a positive diagnosis. Prolonged alcoholism sometimes causes a condition so like true paresis as to render an immediate diagnosis difficult. Time alone can make the distinction certain.

Much can be written about the morbid anatomy, but in a paper of this kind it would be out of place. If unaccustomed to examination of brains, one is apt to be surprised, after the marked clinical symptoms, to be unable to find coarse appearances beyond the usual "congestion" or "anæmia," or "hardened" or "softened" conditions, that makes up the knowledge of most of us.

To quote from an article by Dr. Zimmer, there will appear all that is valuable that can be condensed into the space at our command:

"Paresis is essentially an affection of the anterior portions of the cerebrum, of that part which the study of comparative anatomy and anthropology indicate to be the seat of intelligence, and which modern experimental investigation indicate to contain the motor centers. And it is the morbid manifestations of intelligence and of the motor functions occuring simultaneously and progressing correlatively that constitute the essential features of the disease.

"The pathological anatomy consists of changes in the membranes, more marked in the anterior regions, and changes in the cortical and subcortical substances, and chiefly the anterior cerebral convolutions; the minute changes in brain structure are still a matter of controversy." While the manifestations of symptoms are largely due to cortical and cerebral lesions, in many cases there is disease of the cord also.

